# Mr. Lowe on the Use of Musk and Ammonia

**Published:** 1799-11

**Authors:** W. Lowe

**Affiliations:** Stockport


### *To the Editors of the Medical and Physical Journal.*

GENTLEMEN,

IN the sixth number of the Medical Journal, your correspondent, Mr. SIMMONS, has informed you, that the late Dr. DARBEY was the first person who discovered the efficacy of musk and salt of hartshorn in gangrene and sphacelus. I have already observed to you, that Mr. WHITE, of Manchester, was author of the tract to which you allude in the fifth number of the same Journal, and, as I believed, the first practitioner who had noticed the good effects of the medicine there spoken of. I have but lately had an opportunity of referring to Dr. DARBEY's Thesis; but by looking it over, I have fully satisfied myself that Mr. WHITE not only was the first who promulgated an account of that remedy, but that he also was the first that discovered its efficacy in those affections; and, by permitting me to make a few extracts from each of the publications in question, I think my persuasion will not appear to have been hasty, unless we are not allowed to credit the assertions of Mr. WHITE himself, whose respectability as a gentleman, and whose reputation as a practitioner, the medical world hath long acknowledged. In the advertisement prefixed to his pamphlet, Mr. WHITE says,—"The following observations were made in 1783, but the author "was prevented publishing them, by many professional engagements "and other avocations. They have now been revised, and, though "many cases have since occurred in his practice, and been attended with "the same success, no notice has been taken of them. On dubious "points, too many cases can hardly be produced; but confirmed as he "is of the facts which he has to relate, he considered it as totally un-"necessary to multiply them, especially as they will receive additional "strength from a Thesis, which his worthy friend, Mr. DARBEY, late "apothecary and house-surgeon to the Manchester Infirmary, intends to

"support

" support for a degree in medicine, and which will probably be sub-

" mitted to public inspection." In the pamphlet, p. 11, he says,—

" The remedy I have to lay before the public, is a large and frequent-

" ly-repeated dose of musk and salt of hartshorn, the use of which,

" like that of opium by Mr. Pott, *I discovered rather unexpectedly and*

" *by accident.*" Page 17, he observes,—When I first employed this

" medicine in the complaints to which this pamphlet relates, it was not

" from any expectations, I must own, of stopping their immediate

" progress, but merely to combat disagreeable symptoms, such as the

" singultus, subsultus tendinum, and other convulsive spasms. I soon

" found it not only removed these unpromising appearances, but also

" procured ease, sleep, and a gentle diaphoresis, whilst, at the same

" time, the mortification regularly stopped. The circumstance struck

" me, but I scarcely durst flatter myself the stoppage of the complaint

" itself, in the first instance, was owing to the medieine, till, from re-

" peated trials of it, I observed the same uniform effects. In most of

" the cases in this species of mortification, that have fallen under my

" practice, it has succeeded to the utmost of my wishes, viz. when ac-

" companied with or occasioned by convulsive spasms, or arising from

" local injury, producing irritation." He relates four cases, in which the bark, and other medicines, were freely employed without advantage, when the patients were restored by the use of musk and volatile alkali. The second case, as it is short, and also as it was the first in the Infirmary, where the plan now under our notice was adopted, I will extract.——"James Ogden, of Manchester, aged forty-six, was, on

" the 6th of March, 1780, received into the Manchester Infirmary

" for a compound fracture of the leg: It soon became inflamed; swell-

" ed considerably, with much tension; shewed little signs of digestion;

" and, on the fifth, began to grow livid. The bark was given in con-

" siderable quantities; but, notwithstanding the use of it, the livid

" parts were completely mortified. His pulse beat 140 in a minute;

" his skin was hot and dry; and his tongue without moisture, brown

" and hard. A singultus and subsultus tendinum came on; he had a

" wildness in his looks, succeeded by a delirium. On the eighth from

" the accident, I directed him to take a bolus, containing 10 grains of

" musk with 10 grains of salt of hartshorn, made up with conserve of

" roses, every three hours. He slept better the following night than

" he had ever done since his misfortune, though he had no opiate; and

" the next morning he was in a breathing sweat. His symptoms gra-

" dually abated, but he continued the use of the bolus till he had taken

" two ounces and a half of musk, and as much salt of hartshorn. The

" mortification

"mortification stopped, and the dead parts separating from the living, "came away in large sloughs; but there was such a considerable loss of "substance, that he was not able to leave the Infirmary till the 11th of "December, upwards of nine months from the accident, when he was "discharged with a very useful leg." Allow me now to observe, that Dr. DARBEY graduated at Glasgow, in April 1790, and his Thesis was entitled,—"*Dissertatio Medica quædam de moschi et salis alk. volat.* "*usu, in febre nervosa et gangræna, proponens.*"—He has mentioned only one case of nervous fever, and that is without either date or the name of the person; and, indeed, it could not have affected this enquiry had it been otherwise. The cases of gangrene are related more methodically, and are three in number: The first, page the 15th, was that of Mr. P. who was seized in the month of October 1781, with an erysipelatous inflammation of both legs, and one of them mortified. Mr. DARBEY proposed musk and salt of hartshorn, to the gentlemen who were concerned for the patient, in doses of 10 grains each, three times a day, which he said he had seen of service in cases not much unlike this. After observing that a variety of remedies had been used without effect, he proceeds, page 16,—"*Hoc tempore primum forte hujusce* "*casus historiam mecum communicarunt medici solertes Dr.* HOULSTON, *et* "*Dom.* PARK, *qui medicina probe imbuti, plurima remedia incassum ægro* "*præceperant. Signa, nempe, singultum, tendinum subsultum, somnolen-* "*tiam, torporem, pulsus debiles, delirium sub noctem similiaque mecum in* "*animo volvens, proximo die medicis illis, moschum et salem cornu cervi* "*conjunctos, quos in exemplis non multum hujus disparibus proficere antea* "*videram ægro adhibendos proposui. Aurem facilem præbuerunt propositaque* "*acceperunt generosi supra dicti. Remedii utriusque grana decem ter in die* "*adhibita sunt. Eventum ipsis verbis Dom.* PARK *auscultas, quæ, ex* "*epistola ad Dom.* WHITE, *Mancunii chirurgum, missa, transcribenda cu-* "*ravi. Post signorum quæ paulo supra comprehendi, relationem, progre-* "*ditur Dom.* PARK."——Here follows the event of the case, in a letter from Mr. PARK to Mr. WHITE, which was in favor of the medicine. At the same time he remarks, that it was had recourse to in consequence of a suggestion from Mr. DARBEY, then apothecary at the Infirmary. It does not appear, from the Doctor's account of this case, that he ever saw the patient, nor is it probable he should, as he was not permitted to have any private practice; but this recommendation of the medicine was in a conversation he had with Dr. HOULSTON and Mr. PARK. The next case which he relates, page 18th, was that of J. Hurst, who was brought into the Manchester Infirmary on the 16th of March, 1789, for a fracture of the os humeri; the arm mortified: He took, with success, musk and volatile volatile alk. of each 10 grains, in a bolus, every three hours. The third caſe is much like the ſecond, both as to the complaint and reſult. Theſe are all the caſes the Dr. has related of the employment of this remedy in gangrene. The firſt caſe Mr. White gives us, and in which the diſcovery appears to have been made, was in Sept. 1778. The ſecond is that of a patient of his in the Infirmary, March, 1780. Now, by the nature of his office, as Apothecary to the charity, Dr. Darbey muſt have compounded the medicine which Mr. White preſcribed; and, as houſe-ſurgeon, he muſt have attended the patient: he, therefore, no doubt, had noticed the effect of the medicine in Ogden's caſe, which happened long before he ſuggeſted the uſe of it to Dr. Houlston and Mr. Park; beſide, the agreement in proportion, quantity, and the mode of exhibition, ought to be noticed.—Mr. White's firſt caſe was in 1778; Dr. Darbey's firſt caſe in 1781.—Mr. White claims the diſcovery, in poſitive words, page 11th, and is not contradicted by Dr. Darbey; there is, however, an apparent incongruity in two paſſages of the Theſis: Page the 2d, he ſays,—"*Nemo autem, quantum in memoria nunc teneo,* "*ſuum animum directo ad effectus bonos, quos, in febre typhode atque membris* "*gangræna affectis adhibita, præſtiterunt alkali volat. et moſchus, hactenus* "*attendit*".——Page 25,—"*Hæc exempla ſunt pauca ex multis quæ* "*proferre magnam ad utilitatem moſchi et alkali volat. in febribus invete-* "*ratis et gangræna adhibitorum, confirmandam facile poſſum. Quicunque* "*vero hujuſcemodi plura videre cupiat, tractatum de partibus gangræna af-* "*fectis earundemque curatione, a viro celeberrimo Carolo White armigero,* "*noſocomii Mancunienſis chirurgo, ſocietatis regiæ Londinenſis ſocio, &c. &c.* "*cito edendum, conſulat.*"

I have lately had many opportunities of exhibiting digitalis in hooping-cough, but the length of the above addreſs will not allow me to take up any more room in your Journal; I ſhall therefore conclude with obſerving, that in ſome of the caſes its good effects have ſurpriſed me; at the ſame time, from experience, I can aſſure you, that Dr. Maclean's obſervations, reſpecting the periſhableneſs of digitalis, cannot be too much attended to, as I have frequently been much deceived by it, in conſequence of that quality. If they appear to be worthy of your attention, I will ſend you more particularly my obſervations on its operation.

I am, Gentlemen,

With much reſpect,

Your obedient ſervant,

Stockport, *Sept.* 14. W. LOWE.

